# Artificial Intelligence-Based Ensemble Learning Model for Prediction of Hepatitis C Disease

**DOI:** 10.3389/fpubh.2022.892371

**Published:** 2022-04-27

**Authors:** Michael Onyema Edeh, Surjeet Dalal, Imed Ben Dhaou, Charles Chuka Agubosim, Chukwudum Collins Umoke, Nneka Ernestina Richard-Nnabu, Neeraj Dahiya

**Affiliations:** ^1^Department of Mathematics and Computer Science, Coal City University, Enugu, Nigeria; ^2^College of Computing Science & Information Technology, Teerthanker Mahaveer University, Moradabad, India; ^3^Department of Computer Science, Hekma School of Engineering, Computing and Informatics, Dar Al-Hekma University, Jeddah, Saudi Arabia; ^4^Department of Computer Science, Chukwuemeka Odumegwu Ojukwu University, Uli, Nigeria; ^5^Department of Vocational and Technical Education, Alex Ekwueme Federal University Ndufu Alike Ikwo (AE- FUNAI), Abakaliki, Nigeria; ^6^Department of Computer Science/Informatics, Alex Ekwueme Federal University Ndufu Alike Ikwo (AE-FUNAI), Abakaliki, Nigeria; ^7^Department of Computer Science and Engineering, SRM University Delhi-NCR, Sonipat, India

**Keywords:** artificial intelligence, machine learning, hepatitis C, Quest, ensemble learning

## Abstract

Machine learning algorithms are excellent techniques to develop prediction models to enhance response and efficiency in the health sector. It is the greatest approach to avoid the spread of hepatitis C, especially injecting drugs, is to avoid these behaviors. Treatments for hepatitis C can cure most patients within 8 to 12 weeks, so being tested is critical. After examining multiple types of machine learning approaches to construct the classification models, we built an AI-based ensemble model for predicting Hepatitis C disease in patients with the capacity to predict advanced fibrosis by integrating clinical data and blood biomarkers. The dataset included a variety of factors related to Hepatitis C disease. The training data set was subjected to three machine-learning approaches and the validated data was then used to evaluate the ensemble learning-based prediction model. The results demonstrated that the proposed ensemble learning model has been observed ad more accurate compared to the existing Machine learning algorithms. The Multi-layer perceptron (MLP) technique was the most precise learning approach (94.1% accuracy). The Bayesian network was the second-most accurate learning algorithm (94.47% accuracy). The accuracy improved to the level of 95.59%. Hepatitis C has a significant frequency globally, and the disease's development can result in irreparable damage to the liver, as well as death. As a result, utilizing AI-based ensemble learning model for its prediction is advantageous in curbing the risks and improving treatment outcome. The study demonstrated that the use of ensemble model presents more precision or accuracy in predicting Hepatitis C disease instead of using individual algorithms. It also shows how an AI-based ensemble model could be used to diagnose Hepatitis C disease with greater accuracy.

## Introduction

Hepatitis C is a liver condition caused by the hepatitis C virus (HCV), which affects the immune system. The main reason behind the spreading of Hepatitis C is through the blood contact of an infected person. Life-threatening and serious health issues such as Cirrhosis and liver cancer are the result of chronic hepatitis C. Often, the early-stage symptoms or the sickness don't appear in people suffering from chronic hepatitis C. When these symptoms appear in patients, they might often show signs of advanced liver disease (1). Unfortunately, there is still no vaccine available in medical facilities to treat Hepatitis C. By not sharing needles and refraining from behaviors that could lead to the spread of this disease, such as injecting drugs, the most effective strategy to stop HIV from spreading is to avoid it. Treatments for Hepatitis C that have been evaluated and found effective can cure a patient infected with the virus within 8 to 12 weeks if administered on time.

Hepatitis C infections may be considered as acute (short-term) or chronic (long-lasting). If the symptoms last for 6 months, then that patient is considered to be suffering from acute hepatitis. Unfortunately, in more than 50% of the cases, the patient's body becomes unable to clear the virus and hence this acute infection transforms into a chronic disease. According to some researchers' projections, the United States will be free of this chronic Hepatitis C infection by the year 2036. When it comes to better understanding a condition and its prognosis, medical practitioners can use their patient's electronic health information to uncover new findings and trends that might otherwise go unnoticed by physicians. Machine learning classifiers are used in this work to predict the diagnoses of healthy controls and patients who have been diagnosed with hepatitis C. Other viral infections and cancers may benefit from the use of genetic analysis and machine-learning techniques to choose the best course of treatment. The hepatitis C virus can cause substantial liver damage when it infects the liver and produces inflammation. Blood transfusions, injectable drug usage, and sexual behaviors that expose people to blood are the most common ways in which this virus is spread. It is a bloodborne virus.

The acute phase of hepatitis C infection is the first stage of a long-term infection. Hepatitis C is often misdiagnosed due to the lack of symptoms associated with acute hepatitis C infection. Within 1 to 3 months of infection, acute symptoms occur and continue anywhere from 2 to 3 months. It's not usually the case that an acute hepatitis C infection becomes chronic.

Hepatitis C spreads through contact with the blood of someone who has HCV. This contact may be through (2):

- Hepatitis C spreads through sexual transmission.
- Assisting someone who has HIV by sharing drug needles or other drug paraphernalia Hepatitis C is most commonly acquired this way in the United States.
- Getting poked by a needle that was previously used on someone with HCV. The same thing can happen in a hospital or medical facility.
- Using instruments or inks that have not been disinfected after being used on someone with HCV to get tattooed or pierced
- HCV-infected blood or open sores can spread the disease.
- Razor blades and toothbrushes that may have come into contact with someone else's blood should not be shared.
- Being born to a mother infected with the human papillomavirus (HPV).
- Sexual contact with an HCV-infected person that is not protected

In the period before 1992, the common causes of the spread of Hepatitis C were through blood transfusions and transplants of organs. Infections spread by these ways are now extremely rare in the United States, thanks to the widespread use of routine blood supply testing (3). The medical experts have highly recommended the screening of hepatitis C even for people not showing any symptoms of this disease as after analyzing the population the researchers have concluded that people suffering from chronic hepatitis C may doesn't show any symptoms until it causes complications (4).

Artificial intelligence (AI) advancements assist clinicians in providing patients with faster diagnoses and more effective therapy. Artificial intelligence (AI) and human diagnostic efficiency have been compared in studies. AI was found to be just as good at diagnosing as humans, and in some cases better, when compared to less-experienced doctors. In this article, we introduce an artificial intelligence technique based on previously acquired data from a large number of patients (5). The main objectives of this study are given as below:

- O1: Develop a novel Ensemble model of Machine learning algorithms for Hepatitis C prediction.
- O2: Predicate the level of Hepatitis C using machine learning where it plays a significant role in accurately predicting the disease in different age groups.
- O3: Implement Ensemble model to Hepatitis C prediction and compare it to existing model.

The objective O1 is being designed for predicting Hepatitis C. Moreover, for finding the accurate prediction of Hepatitis C, the objective O2 is planned. Objective O3 will be fulfilled by applying the Ensemble model to predict Hepatitis C. The results are then compared with existing Machine Learning algorithms. The main output of these objectives is predicting Hepatitis C disease more accurately.

The remainder of this paper is organized as follows: Section Related Work reviews the related work in the body of literature. Section Materials and Methods discusses the data and materials used followed by section Methods which explains the theory behind the algorithms. Section Results and Discussion presents and analyzes the experimental results. Finally, the paper concludes and proposes future work in section Conclusion.

## Related Work

To capitalize on this hepatitis information base, scientists utilized a fleeting reflection technique. We offer novel ideas and strategies for abstracting momentary changed and long haul changed tests in light of hepatitis hidden data and information investigation. Information deliberation empowers us to utilize a wide scope of AI methods to distinguish new and intriguing data for clinical doctors (6–9).

Utilizing AI draws near, Mahmoud ElHefnawi et al. (10) fostered an impressively precise prescient model for Egyptian patients' reactions in view of their clinical and biochemical information. The CART characterization calculation was used for choice trees (DTs). Pruning levels of 9, 11, 13, and 17 were examined, as were hubs going in number from 45 to 61 in every one of the six DTs. Hematology action record and fibrosis, viral burden and Alfa-feta protein and egg whites were the most measurably huge instructive parts of the 12 elements in this examination. At last, a 20% test set was utilized to confirm the models. The ANN and DT models have the most noteworthy and middle exactness of 0.76 and 0.69, separately, and 0.80 and 0.72. This test had an exactness of 0.95 percent, and an explicitness of 0.89 and 0.89%. We've reached the resolution that choice trees are more exact at anticipating reaction and can be utilized to direct patients toward the most ideal course of treatment choices.

The essential justification behind liver transplantation in Latin America is hepatitis C disease. Long haul results, for example, demise are further developed when hepatitis C is treated in contaminated patients. Almost 50% of patients treated with pegylated interferon and ribavirin had a drawn out viral reaction. Over the most recent couple of years, new meds have been fostered that have drastically worked on long haul viral reaction, making triple treatment the norm of care. People with chronic hepatitis C now have a place to go in Latin America thanks to these guidelines.

Using a virtual screening (VS) strategy, Wei et al. (11) discovered new HCV NS5B polymerase inhibitors using a combination of irregular backwoods (RB-VS), e-pharmacophore (PB-VS), and docking techniques. A sum of five mixtures were picked for extra enemy of HCV movement testing and cell cytotoxicity examines from the last hit list. Hindrance of NS5B polymerase by each of the five mixtures was viewed as at IC50 values going from 2.01 to 23.84 M, and hostile to HCV action at EC50 values going from 1.61 to 21.88 M. All compounds, except for compound N2, showed no cell cytotoxicity (CC50 > 100 M), albeit compound N2 showed feeble cytotoxicity at a CC50 worth of 51.3 M. There was a particular file of 32.1 for N2's HCV antiviral action, making it the most intense. NS5B polymerase inhibitors could be created from the five hit compounds with novel platforms that have been distinguished.

Hepatitis C infection (HCV) habitually shapes a constant contamination, which is regularly asymptomatic in the beginning phases of the illness. There are as of now no symptomatic models for deciding if a HCV contamination is ongoing vs. persistent. In light of HCV's inclination to mistake in replication, every tolerant has an assorted assortment of hereditarily unmistakable HCV strains. Therefore, it is for the most part accepted that the degree of intra-have HCV heterogeneity develops over the long haul. Because of elements, for ex-ample, particular breadths and negative choice that happen during persistent disease (2, 12), fundamental measurements for evaluating hereditary heterogeneity are not ex-act enough for HCV contamination organizing in light of the intricacy of the primary improvement of HCV populaces inside has.

In a revelation dataset (*n* = 499) of hepatitis B infection (HBV) patients, Wei et al. (13) constructed and contrasted AI draws near and the FIB-4 scoring. The HBV dataset (*n* = 86) was utilized to test the models' presentation. To test the relevance of these models, we applied them to two separate datasets of hepatitis C (HCV) (*n* = 254 and 230). Angle helping (GB) reliably beat FIB-4 scores (p b.001) and different methodologies in the revelation information for the expectation of cutting edge HF and cirrhosis. HBV-approval information showed that the GB model had a region under col-lector working trademark bend (AUROC) that was 0.918, though the FIB-4 model had a region under beneficiary working trademark bend (AUROC) of 0.841. Two HCV datasets utilized GB-based forecast and higher shorts for both GB and FIB-4 scores were important to accomplish equivalent explicitness and awareness, yet the GB-based expectation actually performed well. A correlation of the GB-based forecast technique to FIB-4 in HBV and HCV partners involving different end values for various etiological groupings showed non-stop upgrades comparative with FIB-4. LiveBoost, an easy to understand web stage, empowers our expectation models to be utilized in clinical examination and applications with practically no limitations.

Patients with ongoing hepatitis C were isolated into two gatherings in light of their METAVIR scores: those with gentle to direct fibrosis (F0–F2), and those with cut-ting edge fibrosis (F3–F4). Progressed fibrosis hazard expectation models in view of choice trees, hereditary calculations, molecule swarm advancement, and multistraight relapse calculations have been made. The proposed models were assessed utilizing collector working trademark bend examination. There were genuinely huge relationships between's cutting edge fibrosis and age, platelet count, AST, and egg whites. With an AUROC of 0.73 to 0.76 and a precision of 66.3 to 84.4 percent, the AI calculations had the option to foresee moderate fibrosis in HCC patients. Ends: Alter-native strategies, for example, AI, could be used to estimate the probability of cutting edge liver fibrosis because of constant hepatitis C disease.

As per Cai et al. (14), an outrageous learning machine was prepared to anticipate the fibrosis stage and aggravation action grade of constant hepatitis C utilizing serum lists information from patients to foster a programmed determination framework for persistent hepatitis C. For instance, the basic design and fast estimation speed of the outrageous learning machine make this independent finding framework work well. Serum markers are utilized to survey the proposed robotized determination framework for ongoing hepatitis C. For the finding of ongoing hepatitis C fibrosis stage and provocative movement grade, trial information show that the recommended method beats current baselines.

Utilizing Non-direct Iterative Partial Least Squares, Self-Organizing Map strategy, and troupes of Neuro-Fuzzy Inference System, Mehrbakhsh estimate the hepatitis infection. Likewise, we utilize choice trees to pick the most important highlights in the exploratory dataset. Utilizing a genuine world dataset, we put our procedure under serious scrutiny and contrast the outcomes with those of prior investigations. Utilizing the dataset, we observed that our strategy outflanked the Neural Network, ANFIS, K-Nearest Neighbors, and Support Vector Machines. As a shrewd learning framework for hepatitis sickness diagnostics in the medical care industry, this innovation has incredible guarantee.

Utilizing an AI approach, Ahammed et al. (15) fostered a calculation that can precisely group the periods of liver sickness in hepatitis C tainted patients. They utilized the UCI AI archive to acquire instances of liver fibrosis sickness in Egyptian patients. Manufactured minority oversampling approach has been utilized to expand the quantity of engineered patients to keep an even conveyance across all classifications. They then, at that point, utilized an assortment of element choice methods to decide the main hepatitis C viral characteristics in this dataset. Classifiers have been utilized to separate patients into bunches in view of whether their HCV cases are adjusted essential, include picked essential, or essential. In the wake of examining the information, KNN ends up as the winner, with a precision pace of 94.40%. Hepatitis C infection irresistible sickness has profited from this present review's discoveries.

NGS (cutting edge sequencing) is a usually involved strategy for delivering top caliber, profound, and proficient arrangement information. The pre-S area of the HBV genome was sequenced in 139 people, including 94 HCC patients and 45 constant HBV (CHB) patients, utilizing NGS innovation. We made two various types of information-al indexes. To begin with, we used an essential nearby arrangement search instrument (BLAST) to plan every NGS short read and convert every arrangement into an amino corrosive by DNA codon table for the information on amino corrosive event recurrence. The info highlights are the Shannon entropy-based event frequencies of 20 major amino corrosive.

## Materials and Methods

### Dataset

The dataset used in this research work was fetched from the source- Kaggle. The data set includes laboratory values of blood donors and patients suffering from Hepatitis C along with their demographic factor values such as age. The data was collected from UCI Machine Learning Repository: https://archive.ics.uci.edu/ml/datasets/HCV+data.

Except Category and Sex values, all other attributes are numerical. For classification, the target attribute is Category (2): blood donors vs. Hepatitis C patients [including its progress (“just” Hepatitis C, Fibrosis, Cirrhosis)].

### Methods

#### Data Processing

In order to transform the fetched raw data in useful and highly efficient format, certain data pre-processing techniques are employed. At this stage, different types of functions are implemented so as to find the missing values, outliers, redundant and skewed features (16).

#### Missing Values

Once the above pre-processed data is loaded, a function is employed to find the missing values in relation to each feature (17). The term “missing data” refers to values that aren't available, and that would be relevant if they were observed. For example, a data input error or incomplete file might cause a data set to be blank or out of sequence. Missing data is a common occurrence in real-world datasets. For analysis and modeling purposes, you must first convert data with missing data fields. This, too, might be more art than science, as is the case with many other parts of data science. Having an understanding of the data and the context in which it originates is critical. A setback isn't always a setback when you have missing numbers in your data. Although the model is lacking some information, this is a chance to do some feature engineering to help it make sense of what's been omitted. Automated detection and remediation of missing data is possible thanks to machine learning techniques and software packages.

#### Outliers Data

After the above pre-processing stage, a certain category of data is referred to as noisy data if it may be corrupted, distorted, or cannot be interpreted. This kind of data may have originated from improper procedures or wrong data collection. However, it can be handled by implementing the methods such as regression, clustering or binning (18–20).

#### Multilayer Perceptron

**Figure 1:**
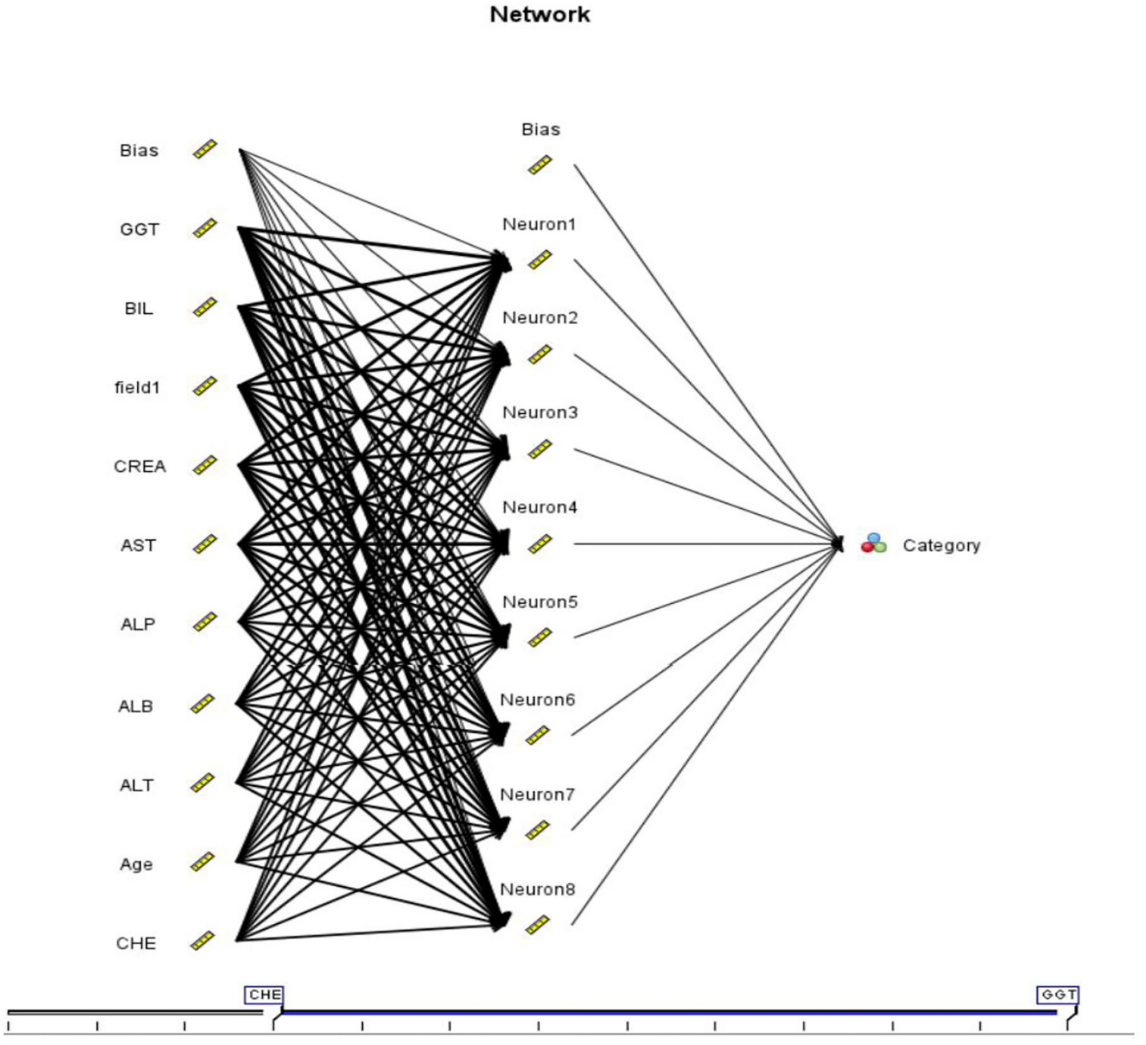
MLP Diagram.

The feedforward artificial neural network known as a multilayer perceptron (ANN). Multi-layer perceptron (MLP) networks are commonly referred to as feedforward ANNs, but the word is also used more specifically to describe a specific type of ANN with many layers of perceptrons (with threshold activation). The input layer transmits the signal to be processed. Prediction and categorization are under the purview of the output layer. There may be any number of hidden layers between the input and output levels in the MLP's computational engine (11, 14, 21) as seen in Figure 1.

**Figure 2:**
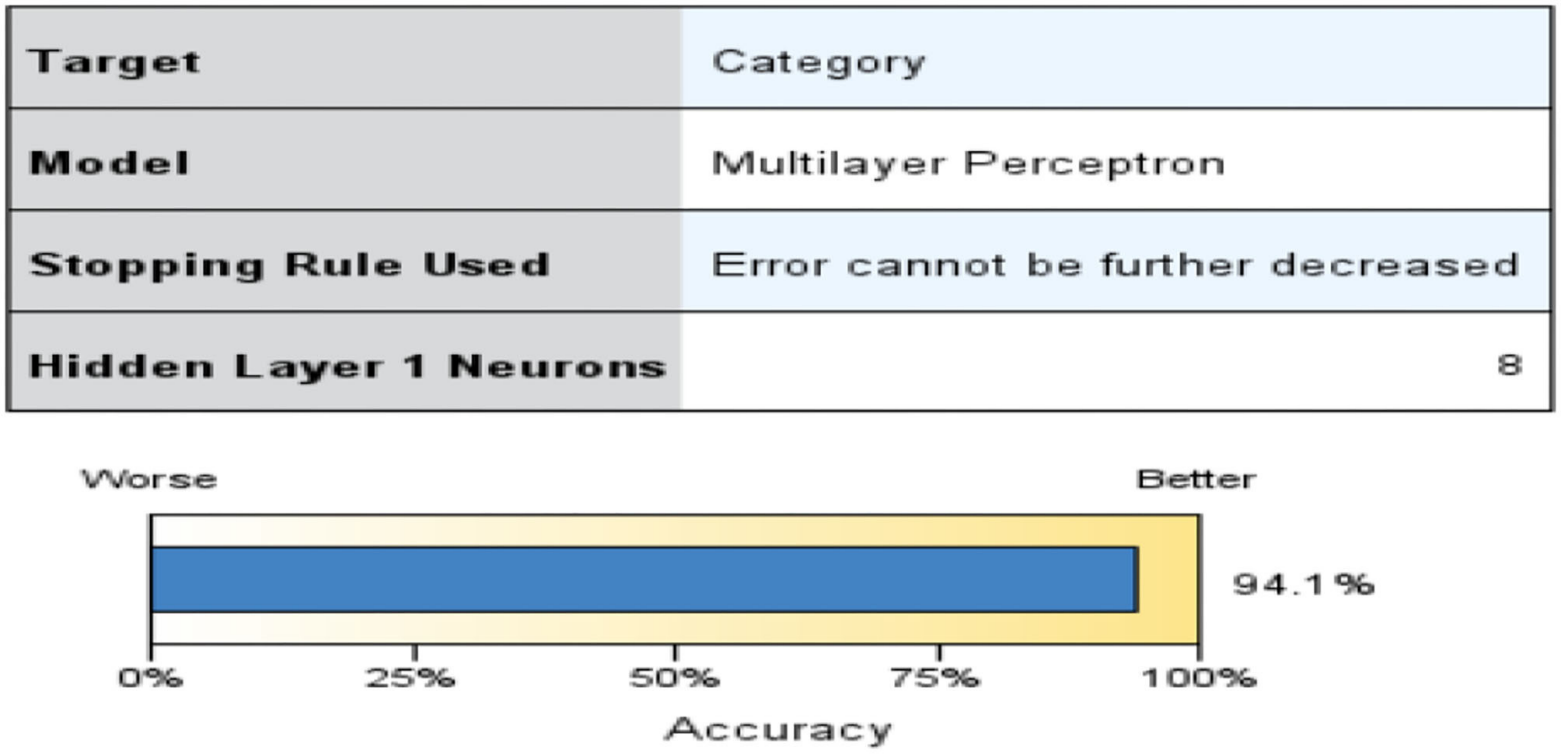
MLP Accuracy.

For the back propagation process to work, it needs to traverse two different layers of a network in both directions. Pattern or input vector are applied to the input layer, and this effect propagates across multiple layers and generates an output vector in the forward pass The weights of the synapses in the network remain constant during this operation. The weights vary during the backward pass because of the mistake correction rule. Comparing the output signal's present state to the desired state is done (13, 22) as seen in Figure 2.

**Figure 3:**
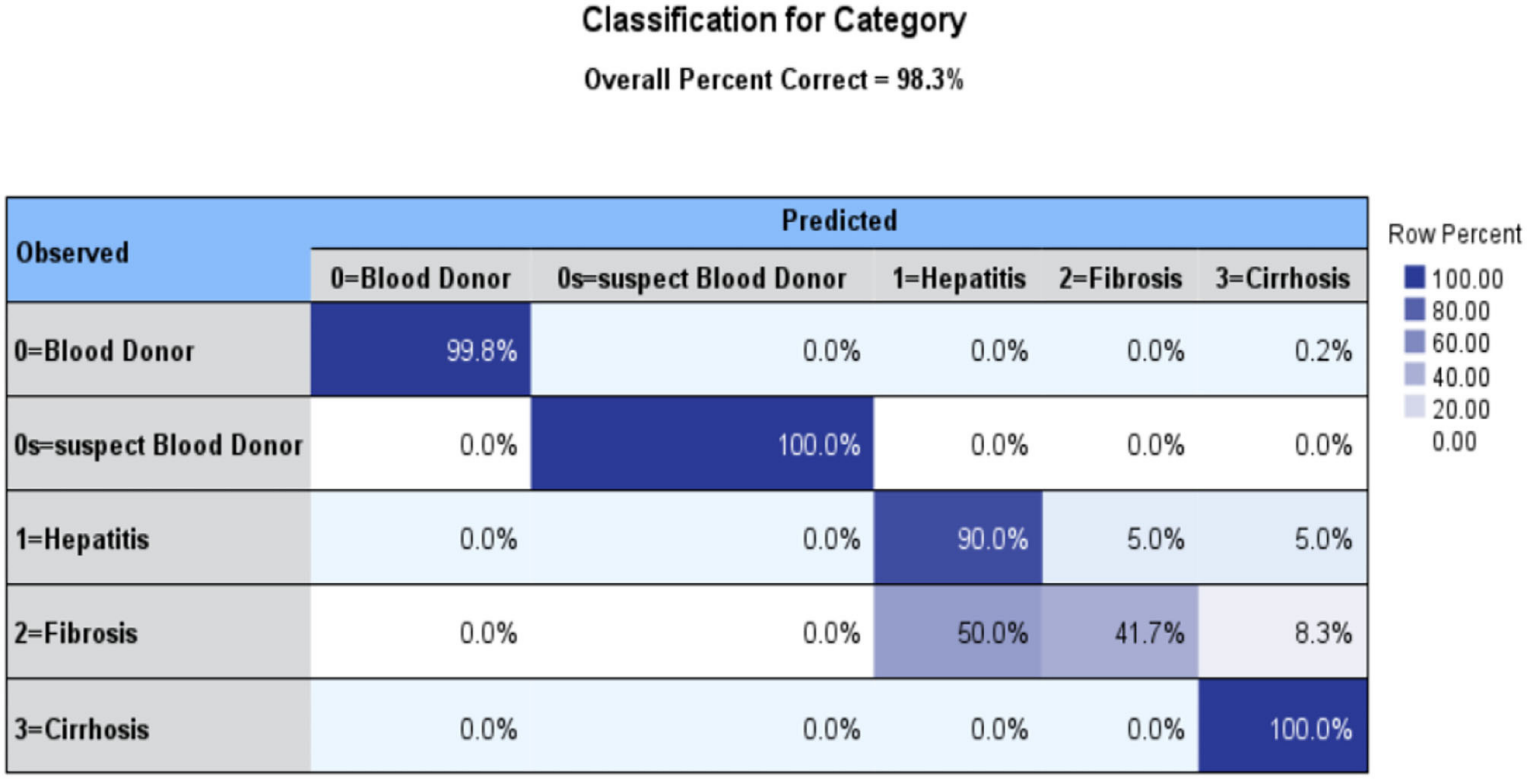
MLP Classification for Category.

Using MLPs, it is possible to approximate any continuous function, including those that cannot be separated linearly. Pattern classification, recognition, prediction, and approximation are the most common uses of MLP as seen in Figure 3.

#### Bayesian Network

As an alternative, developing a model that preserves known conditional dependence between random variables and conditional independence in all other situations. In a graph with directed edges, Bayesian networks represent the probabilistic graphic representation of a system's known conditional dependence. The Bayesian network seen in **Figure 6** depicts the structure of the problem domain. In order to identify hepatitis illness, the network models a variety of factors, including certain symptoms and a small number of disorder nodes (10, 15, 23–33).

**Figure 4:**
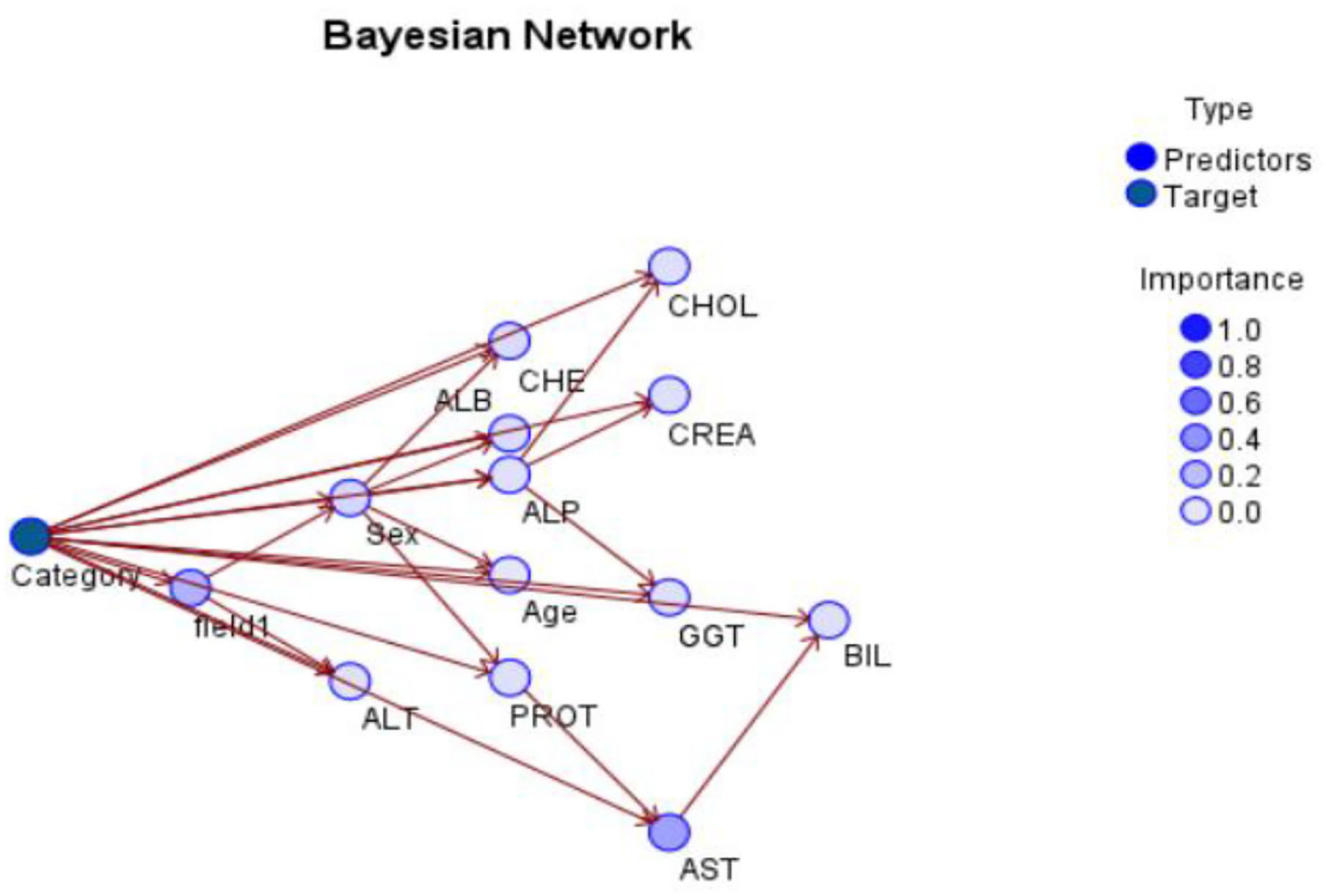
Bayesian network for current problem.

**Figure 5:**
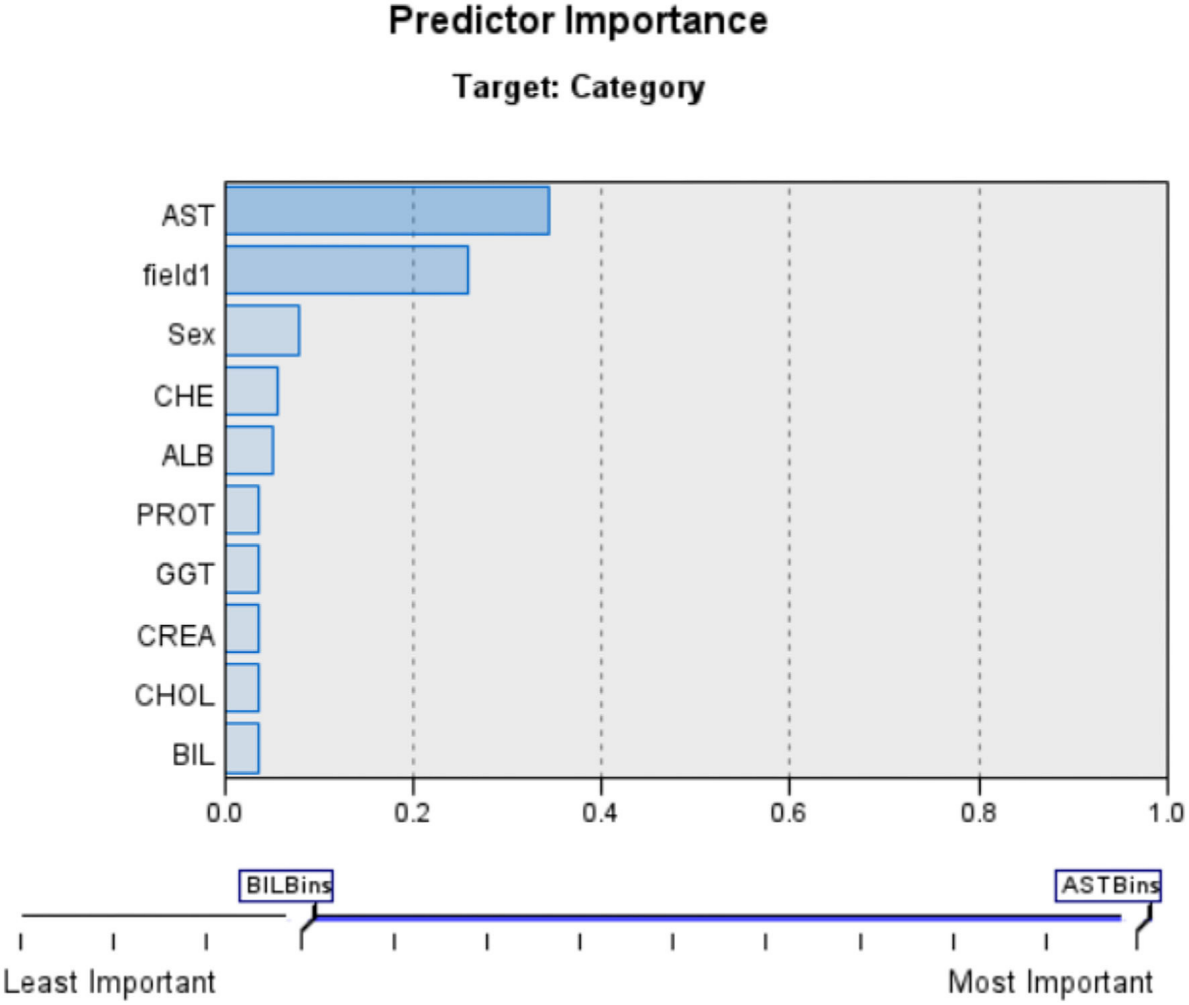
Bayesian network Accuracy.

Hepatitis C is more likely in those who have jaundice, and vice versa, as shown by the arcs between the two nodes: jaundice raises the likelihood of Hepatitis C, and vice versa. The model's layout serves as an illustration of the causal links between the many elements involved in the diagnostic process. Figures 4, 5 shows the Bayesian network for the problem under study in this paper and its accuracy.

**Figure 6:**
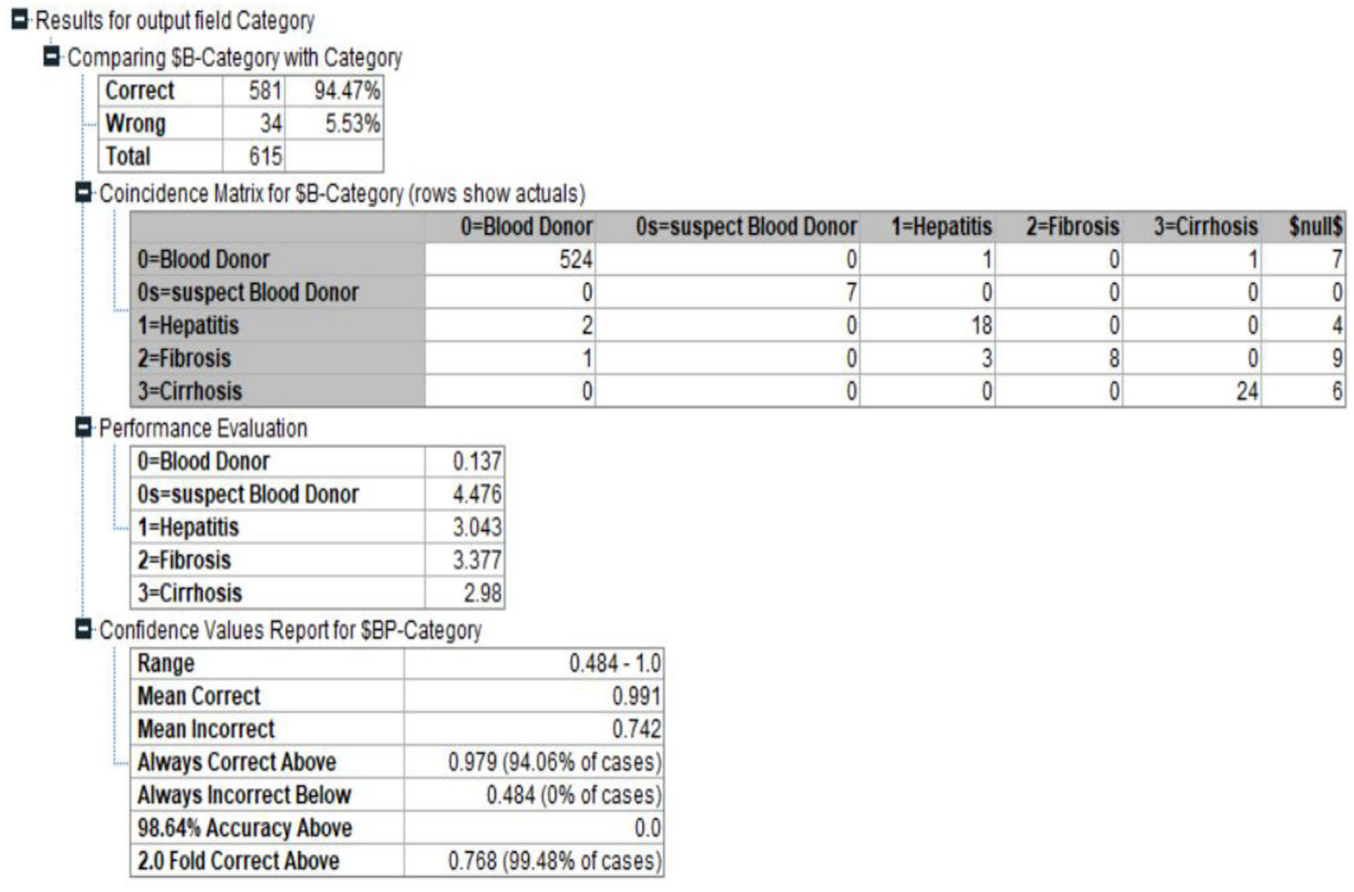
Bayesian network Classification for Category.

In creating the framework, we drew inspiration from medical fiction, conversations with herpetologists, and the model's numerical elements, including as Hepatitis patients' medical records are mined for prior and conditional probability distributions. Figure 6 shows the Bayesian network Classification for Category.

#### Quest

**Figure 7:**
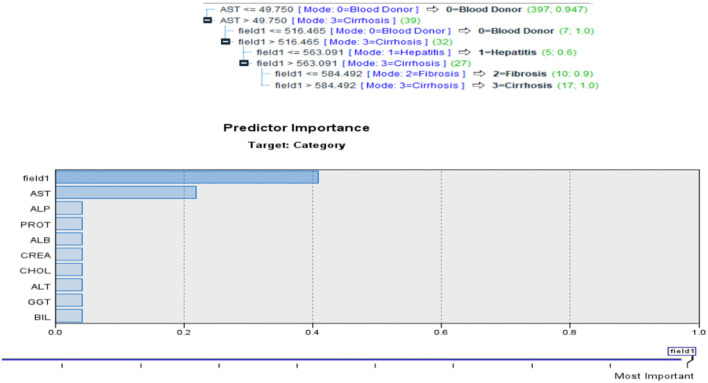
QUEST for current problem.

Classification systems and prediction algorithms can be built using decision tree approach, which is commonly used in data mining. Branch-like segments of a population form an inverted tree with a root node, inner nodes, and leaf nodes. Rather than relying on a complicated parametric framework, the method uses a non-parametric approach to deal with large and complex datasets. When the sample size is large enough, training and validation datasets can be segregated from one other. QUEST evaluates a node's predictor variables using a set of criteria based on significance tests. Each predictor at a node may only need to be tested once for selection reasons. This method does not analyze splits as thoroughly as either C&RT or CHAID does, nor does it study category combinations as thoroughly as either C&RT or CHAID does as seen in Figure 7.

**Figure 8:**
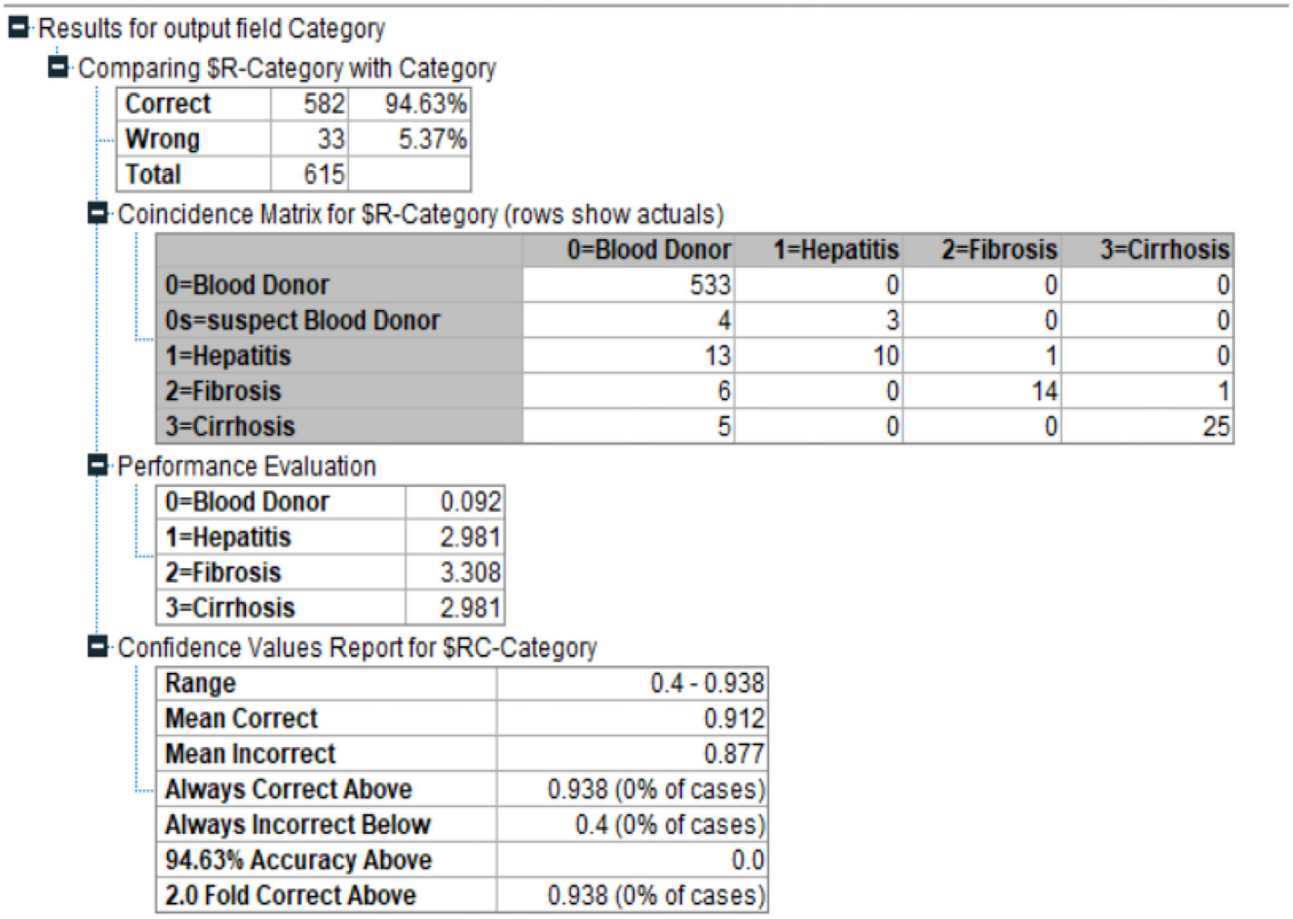
Quest Classification for Category.

This helps to speed things up. The target categories are divided into groups by doing a quadratic discriminate analysis using the specified predictor. When determining the ideal split, this approach is faster than C&RT (Complete and Recursive Search). Quest Diagnostics, a major clinical laboratory test supplier in the United States, provided the data for this study. Also Figure 8 depicts the Quest Classification for Category.

Ordered HCV antibody immunoassay testing and RNA diagnostic tests for de-identified individuals. When available, data on patient gender and age were incorporated into the study. It was concluded that the data obtained from Quest Diagnostics could not be used to identify any particular person. In the event of a positive antibody test result, all specimens will be sent for HCV RNA quantitative testing.

#### Proposed Ensemble Learning Model

Combining predictions from multiple models to improve predictive performance is called ensemble learning. As many ensembles as you like may be created for any predictive modeling challenge, but there are only three methods that dominate the field of ensemble learning. Much to the dismay of many, each algorithm has given rise to an infinite number of sub-algorithms. It's important to understand the three main classes of ensemble learning methods before moving on to the math and programming, so that we can understand the underlying concepts.

##### Typical Ensemble Learning Techniques

Although there are practically infinite ways to accomplish this, there are probably three kinds of ensemble learning approaches that are most frequently studied and employed in practice. Their appeal stems from their ease of use and ability to solve a wide range of predictive modeling challenges. They are:

- Bagging.
- Stacking.
- Boosting.

Each strategy has an algorithm that specifies it, but the success of each approach has crucial to realize that, while these three methods are widely discussed and used, they don't define the scope of ensemble learning on their own.

##### Bagging Ensemble Learning

It is an ensemble learning method that uses bootstrap aggregation or bagging. Bootstrap and aggregation are the two primary components of Bagging, as their names suggest. An unpruned decision tree is almost commonly used to train each model on a separate sample of the same training dataset in this manner. A basic statistic, such as a vote or average, is used to combine the forecasts of the ensemble members. When training the classifiers, we utilize a weak classifier whose decision boundaries vary significantly in response to even small perturbations in our training data. This ensures that the ensemble has a diverse set of classifiers.

When training ensemble members, the preparation of each dataset sample is critical. The dataset is randomly sampled for each model. In this dataset, the examples (rows) are chosen at random, but replacement is performed on them. The bootstrap distribution is used in bagging to generate alternative base learners. To put it another way, data subsets are collected via bootstrap sampling for the purpose of training the foundational learners.

It's called a bootstrap sample. In statistics, it's a method for assessing the statistical significance of a small sample of data. Rather than estimating directly from the dataset, it is possible to obtain a more accurate overall estimate of the desired quantity by doing many bootstrap sampling. Training datasets for many independent predictive models can be built in the same way and used to make predictions. Rather to fitting a single model directly to the training dataset, averaging the predictions from multiple models is often more accurate. Bagging's most important characteristics can be summarized as follows:

- Samples from the training dataset that have been bootstrapped.
- On each sample, unpruned decision trees fit.
- Simple voting or prediction averaging.

In summary, bagging makes a contribution by changing the training data used to fit each ensemble member, resulting in skilled but distinct models.

##### Bagging Ensemble

It's a broad strategy that can be readily expanded. More changes to the training dataset, for example, could be made, the algorithm that fits the training data could be altered, and the mechanism that combines predictions might be changed. This method is used in a number of popular ensemble algorithms, including:

- Bagged Decision Trees (canonical bagging)
- Random Forest
- Additional Trees

Next, let's look into stacking in more detail.

##### Stacking Ensemble Learning

Stacked To find a diverse group of members via generalization or stacking, one can vary the model types that are fit on training data and combine these models' predictions with one another. Stacking is a common technique for teaching a student how to mix up a large group of students. In education, the term “first-level learner” refers to an individual student, whereas “second-level learner” refers to the combined group. Level-0 models refer to the members of an ensemble, while level-1 models describe the model used to integrate the forecasts. The following are a few of the most important aspects of stacking:

- The training dataset has not been modified.
- For each ensemble member, different machine learning techniques are used.
- Using a machine learning model, we can figure out how to combine predictions in the most effective way.

The several machine learning models that make up the ensemble add variety. Using a number of models that are taught or developed in a variety of ways ensures that they make a wider range of assumptions and, as a result, have less related predictions errors (34, 35). In an ensemble method known as “boosting,” the training data is manipulated in an attempt to draw attention to examples that previous models that were fitted to the training dataset mistakenly detected. In boosting, the training dataset for each new classifier is progressively narrowed to examples that prior classifiers had incorrectly classified. The idea of correcting prediction errors is an important characteristic of boosting ensembles. To make sure that the first model's predictions are as accurate as possible, subsequent models are fitted and added to the ensemble in a logical order. Weak learners, or decision trees that only make one or a few judgments, are commonly used to do this.

Data can be weighed to signify how much attention an algorithm should give the model while it is being learnt. The following are a few of the most important aspects of boosting:

- Bias training data in favor of difficult-to-predict examples.
- Using a weighted average of models, combine forecasts.

There have been a few different approaches to making it possible to turn a large number of weak students into a small number of strong ones. The Adaptive Boosting (AdaBoost) method proved boosting to be an effective ensemble strategy for this paper. An algorithm known as “boosting” can predict more accurately.

## Results and Discussion

In this research paper, following machine learning algorithms are being used including:

- MLP
- Bayesian Network
- QUEST

**Figure 9:**
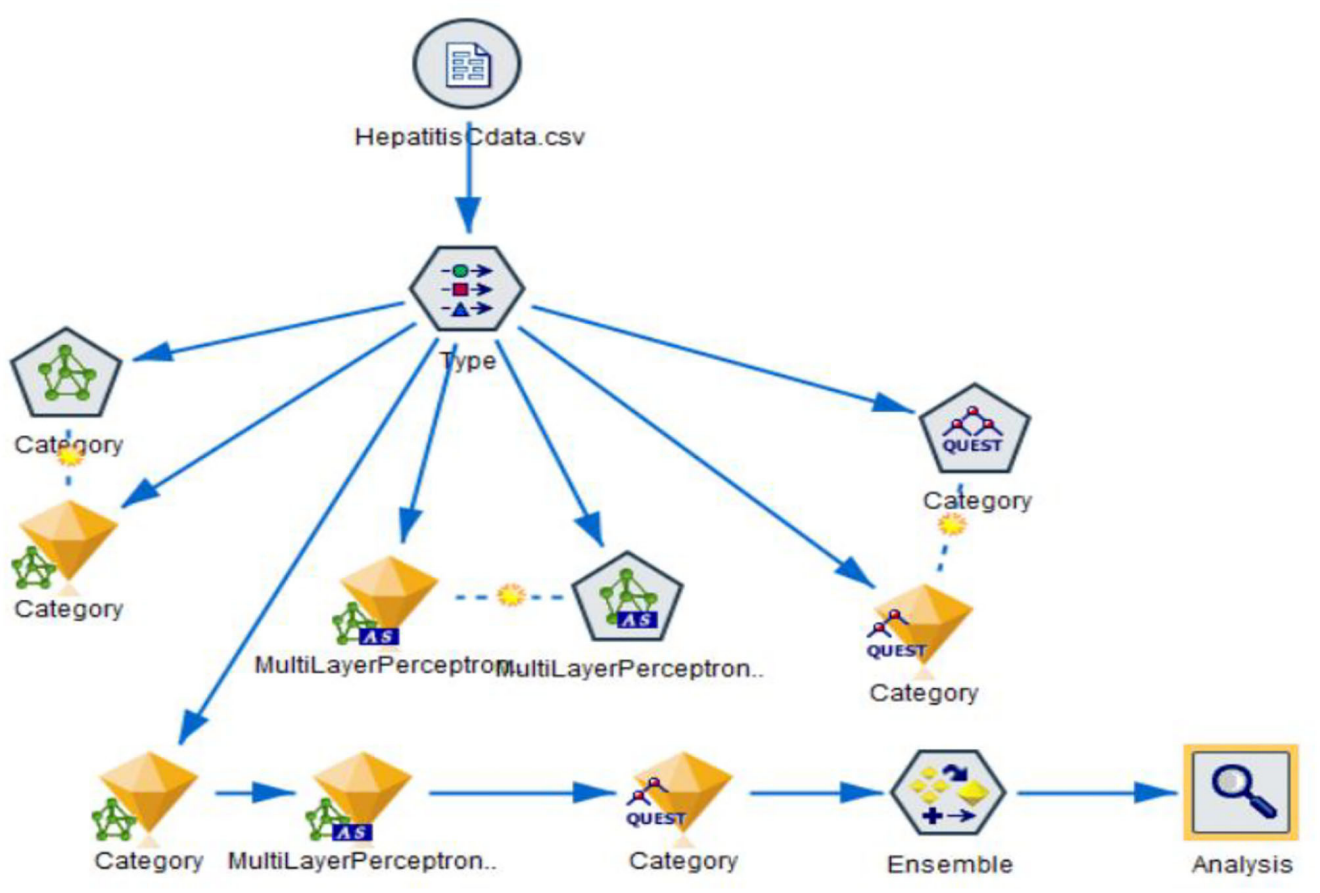
Proposed Ensemble learning model.

**Figure 10:**
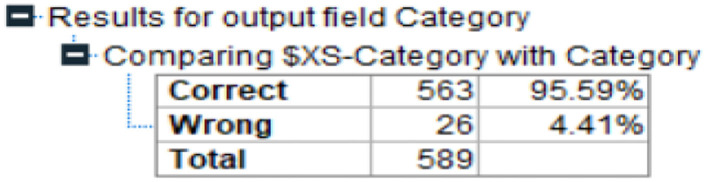
Accuracy level of Proposed Ensemble learning model.

The Figures 9, 10 depicts the working of the proposed Ensemble Learning model. Ensemble node mixes three model nuggets to obtain more accurate predictions than can be derived from any of the individual models (MLP, Bayesian Network & QUEST) (MLP, Bayesian Network & QUEST). By merging predictions from different models, limitations in MLP, Bayesian Network & QUEST models have been eliminated, resulting in a higher overall accuracy. MLP, Bayesian Network & QUEST Models integrated in this manner often perform at least as well as the best of the MLP, Bayesian Network & QUEST models and often better.

**Figure 11:**
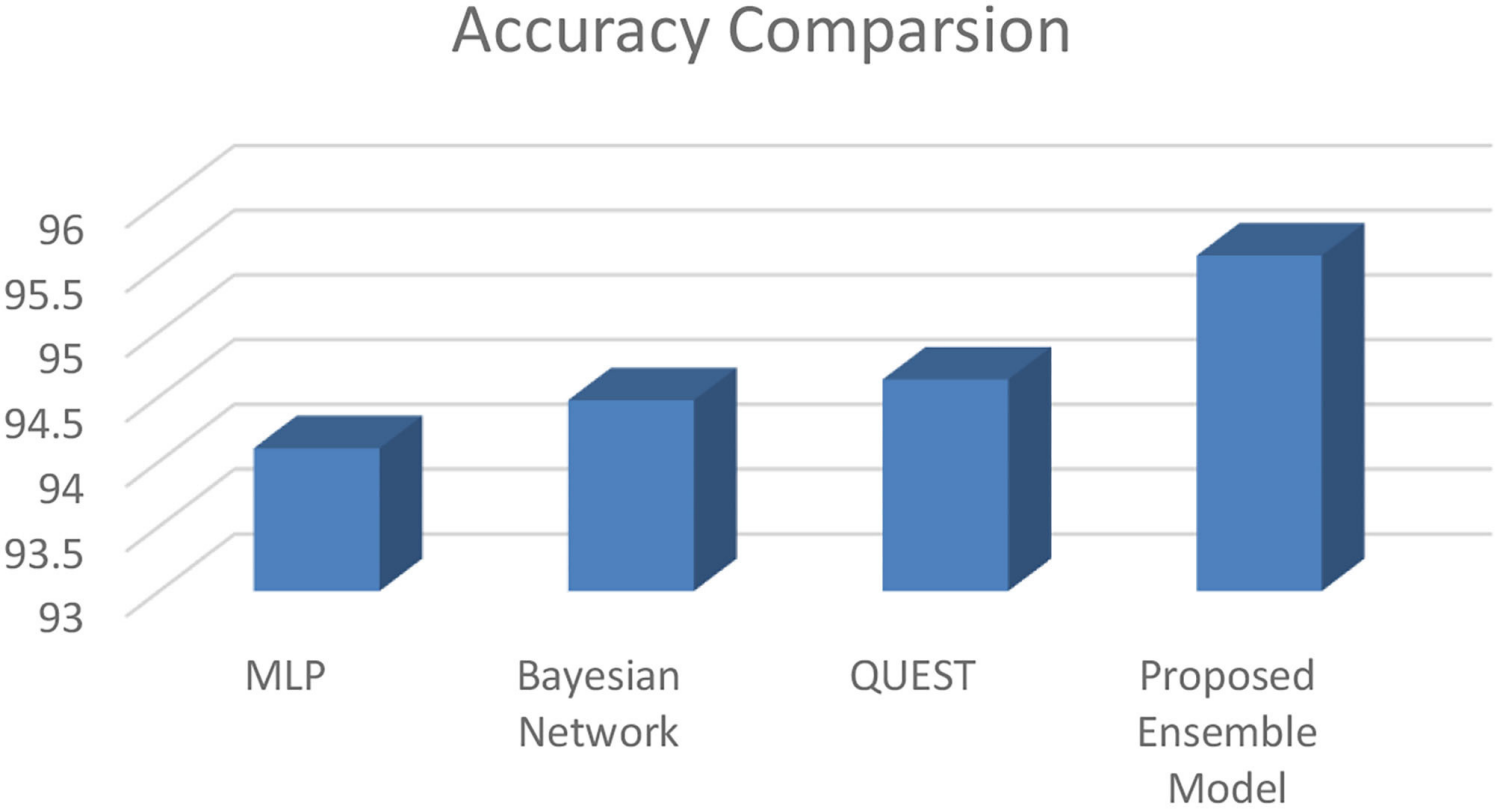
Accuracy comparison.

Table 1 represents the level of accuracy at 95.59%. This value is more efficient to individual machine learning algorithms as shown in Table 1. This is also represented in Figure 11.

**Table 1:** Accuracy comparison.

**S. No**.	**Name of Algorithm**	**Accuracy (%)**
1	MLP	94.10
2	Bayesian Network	94.47
3	QUEST	94.63
4	Proposed Ensemble Model	95.59

Then results of this proposed model indicate that the proposed ensemble model can accurately predict the advanced fibrosis stage in chronic HCV patients. The proposed model offers us 95.9% correct results. We can improve the model's efficiency in the future by adding more patient data. In addition, more liver ailments could be investigated in the future. Figures in the paper appeared in sequence.

## Conclusion

In this research paper, different machine learning algorithms are being applied for predicting advanced liver fibrosis in Chronic Hepatitis C patients. It is observed that individual model is capable of providing the accuracy up to 94.67%. Then the ensemble model including Bayesian network, MLP and QUEST decision trees has been developed.

In designing healthcare systems, innovative techniques that carefully balance public health initiatives with limited resources should be taken into consideration. In order to help individuals who don't have health insurance or are underinsured, there should be an increase in access to healthcare, community outreach, and the growth of telemedicine, including safe laboratory testing.

## Data Availability Statement

Publicly available datasets were analyzed in this study. This data can be found here: https://archive.ics.uci.edu/ml/datasets/HCV+data.

## Author Contributions

All authors listed have made a substantial, direct, and intellectual contribution to the work and approved it for publication.

## Conflict of Interest

The authors declare that the research was conducted in the absence of any commercial or financial relationships that could be construed as a potential conflict of interest.

## Publisher's Note

All claims expressed in this article are solely those of the authors and do not necessarily represent those of their affiliated organizations, or those of the publisher, the editors and the reviewers. Any product that may be evaluated in this article, or claim that may be made by its manufacturer, is not guaranteed or endorsed by the publisher.
